# A data-mining study on the prediction of head injury in traffic accidents among vulnerable road users with varying body sizes and head anatomical characteristics

**DOI:** 10.3389/fbioe.2024.1394177

**Published:** 2024-04-30

**Authors:** Qiuqi Yuan, Jingzhou Hu, Zhi Xiao, Bin Li, Xiaoming Zhu, Yunfei Niu, Shiwei Xu

**Affiliations:** ^1^ School of Mechanical and Vehicle Engineering, Hunan University, Changsha, China; ^2^ State Key Laboratory of Advanced Design and Manufacturing Technology for Vehicle, Hunan University, Changsha, China; ^3^ Suzhou Research Institute, Hunan University, Suzhou, China; ^4^ Department of Oral and Maxillofacial-Head and Neck Oncology, Shanghai Ninth People’s Hospital, Shanghai Jiao Tong University School of Medicine, Shanghai, China; ^5^ Shanghai Motor Vehicle Inspection Certification and Tech Innovation Center Co., Ltd., Shanghai, China; ^6^ Changhai Hospital., Shanghai, China

**Keywords:** anatomical characteristics, head injury, vulnerable road users, traffic accidents, data mining

## Abstract

Body sizes and head anatomical characteristics play the major role in the head injuries sustained by vulnerable road users (VRU) in traffic accidents. In this study, in order to study the influence mechanism of body sizes and head anatomical characteristics on head injury, we used age, gender, height, and Body Mass Index (BMI) as characteristic parameters to develop the personalized human body multi-rigid body (MB) models and head finite element (FE) models. Next, using simulation calculations, we developed the VRU head injury dataset based on the personalized models. In the dataset, the dependent variables were the degree of head injury and the brain tissue von Mises value, while the independent variables were height, BMI, age, gender, traffic participation status, and vehicle speed. The statistical results of the dataset show that the von Mises value of VRU brain tissue during collision ranges from 4.4 kPa to 46.9 kPa at speeds between 20 and 60 km/h. The effects of anatomical characteristics on head injury include: the risk of a more serious head injury of VRU rises with age; VRU with higher BMIs has less head injury in collision accidents; height has very erratic and nonlinear impacts on the von Mises values of the VRU’s brain tissue; and the severity of head injury is not significantly influenced by VRU’s gender. Furthermore, we developed the classification prediction models of head injury degree and the regression prediction models of head injury response parameter by applying eight different data mining algorithms to this dataset. The classification prediction models have the best accuracy of 0.89 and the best R2 value of 0.85 for the regression prediction models.

## 1 Introduction

Among road users (e.g., pedestrians, cyclists, occupants, etc.), pedestrians and cyclists are vulnerable road users (VRU) (Ahmed et al., 2019; Mahmoud et al., 2021; Reyes-Muñoz and Guerrero-Ibáñez, 2022). According to the statistics report (WHO, 2018), the primary cause of the VRU injury in the traffic accident is the frontal collision with the vehicles. The fatality rate of severe head injury in human is very high, with 64% of VRU death due to blows to the head. Therefore, for head protection and the reduction of mortality, precise prediction of VRU head injuries in traffic accidents is crucial.

The influencing factors of head injury in traffic accidents are very complicated (Xiao et al., 2020; Wang F. et al., 2021; Han et al., 2024). In addition to objective factors (e.g., vehicle speed, collision position, collision angle, vehicle type, etc.), the diversity of body sizes will also lead to significant differences in head dynamics (e.g., acceleration, angular velocity, displacement, etc.) (Gao et al., 2021). Moreover, variations in the intricate anatomical structure of the head will inevitably result in variations in the injury response parameters (e.g., von Mises, pressure, strain, etc.) (Shi et al., 2020). The multi-rigid body (MB) model, which can replicate head dynamics responses, and the human finite element (FE) model, which can replicate head injury parameters, have emerged as the primary tools for investigating head injury mechanisms with the advancement of simulation technology (Hu et al., 2021; Larsson et al., 2023; Li et al., 2023; Delteil et al., 2024). However, the current research focuses on the application of the MB models to study the influence of objective factors on the head dynamic response, and there is a lack of detailed research on the mechanism of head injury caused by body size and head anatomical structure. This is partially because the MB models can’t accurately represent the types and severity of brain injury because they only include rigid bodies, hinges, and simplified ellipsoids. Concurrently, despite the fact that the human body FE model includes the detailed human anatomical structure, a single kind of human FE model is unable to explain the impact of geometric anatomy on the response parameters of head injury because there are insufficient samples of detailed head anatomy. Consequently, it is critical to develop personalized VRU models in order to study the impact of body sizes and head anatomical characteristics on the mechanism of VRU head injury.

Additionally, the mechanism of VRU head injury in traffic accidents becomes a high-dimensional problem with various influencing aspects after taking into account objective factors, body sizes, and head anatomy characteristics. The traditional univariate analysis method has some shortcomings in studying the mechanism of head injury, such as low efficiency and being unable to identify the interaction between multiple factors. Artificial intelligence technology has been used to solve the high-dimension problem in data prediction research since the development of data mining technology (Janstrup et al., 2023; Niu et al., 2024). Classical machine learning (ML) algorithms (e.g., logistic regression, k-nearest-neighbors, support vector machines, decision tree, random forests, etc.) have been used to develop a prediction model of human injury degree (Al-Moqri et al., 2020; Mansoor et al., 2020; Liu et al., 2022). At the same time, in order to further explore the interaction relationship between objective factors in traffic accidents and human injury response to improve the accuracy of prediction models, deep learning (DL) method with higher computational complexity (e.g., deep neural network, convolutional neural network, long short-term memory, recurrent neural network, etc.) have been applied to the construction of head injury prediction models (Wang Q. F. et al., 2021). Currently, accident investigation datasets or customized datasets created through simulation are used in the development of head injury prediction models. Nonetheless, accident investigation datasets typically overlook specific head anatomy in favor of concentrating on how external factors affect the severity of head injury. Furthermore, the simulation-based customized datasets only take into account the head’s dynamic reaction factors, which makes them unable to accurately represent the circumstances surrounding actual head injury.

The objective of the present study is to investigate the influences of anatomical characteristics on the VRU head injury in collision incidents and develop a prediction model that can accurately predict it. We built a head injury dataset based on the personalized models that can reflect VRU body sizes and head anatomical characteristics. Subsequently, eight different types of data mining methods were applied to this dataset in order to create prediction models for VRU head injury degree classification and response (von Mises value) regression. Lastly, the capacity of prediction models based on various algorithms for predicting injury was assessed, and related explanations were presented.

## 2 Methods

**FIGURE 1:**
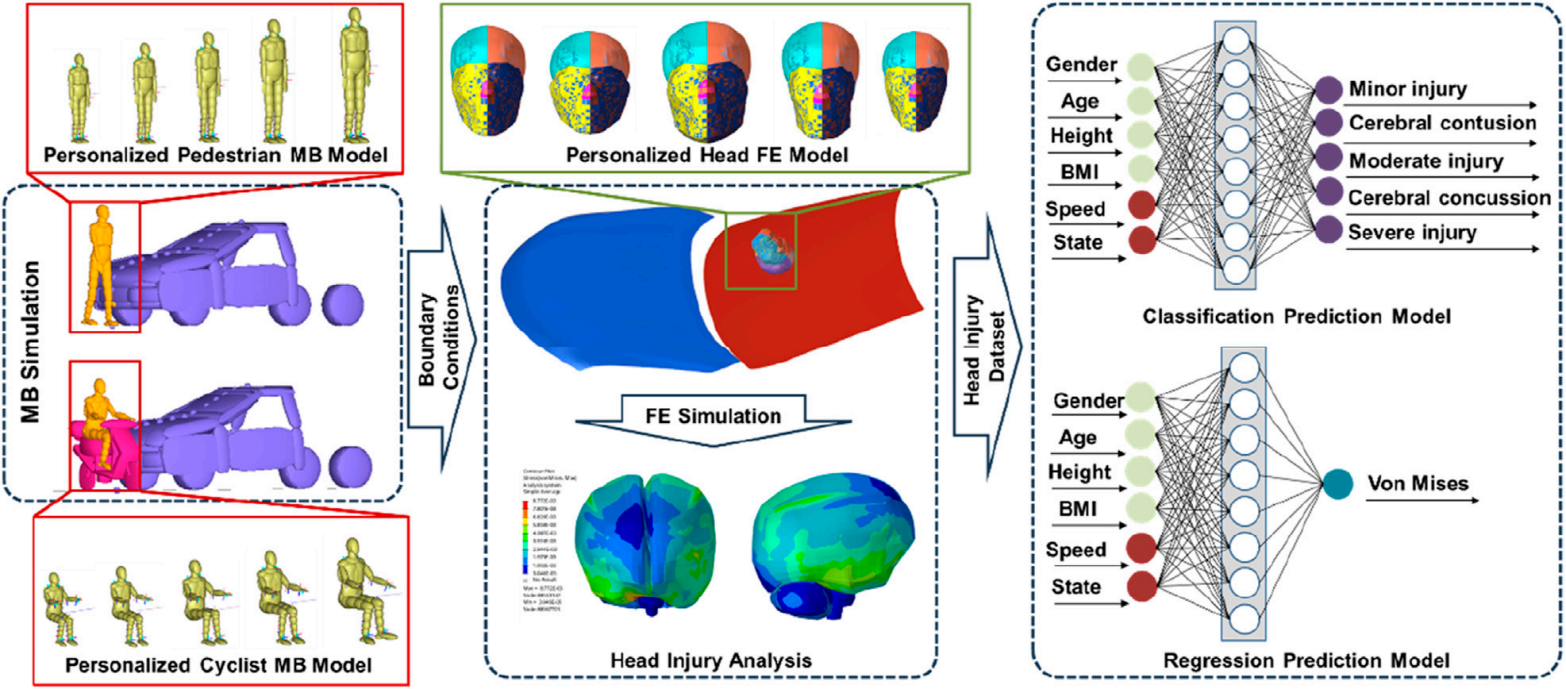
Technical framework for predicting head injury in traffic accidents among VRU with varying body sizes and head anatomical characteristics.

The technical framework of this study is shown in Figure 1. Initially, the personalized human body MB models and the head FE models were developed. The correctness of the models was then confirmed through comparison with a cadaver experiment. These models were then used to develop the VRU—vehicle frontal collision numerical simulation dataset. The regression prediction models of head injury response parameter and the classification prediction models of head injury degree were developed using ML and DL algorithms based on this dataset. The inputs for these prediction models were vehicle speed, traffic participation states, and VRU characteristic factors; the outputs were the head injury degree and von Mises value of the brain tissue. At last, the prediction models undergo verification and evaluation.

### 2.1 Modeling method

#### 2.1.1 Acquisition of head sample data

**FIGURE 2:**
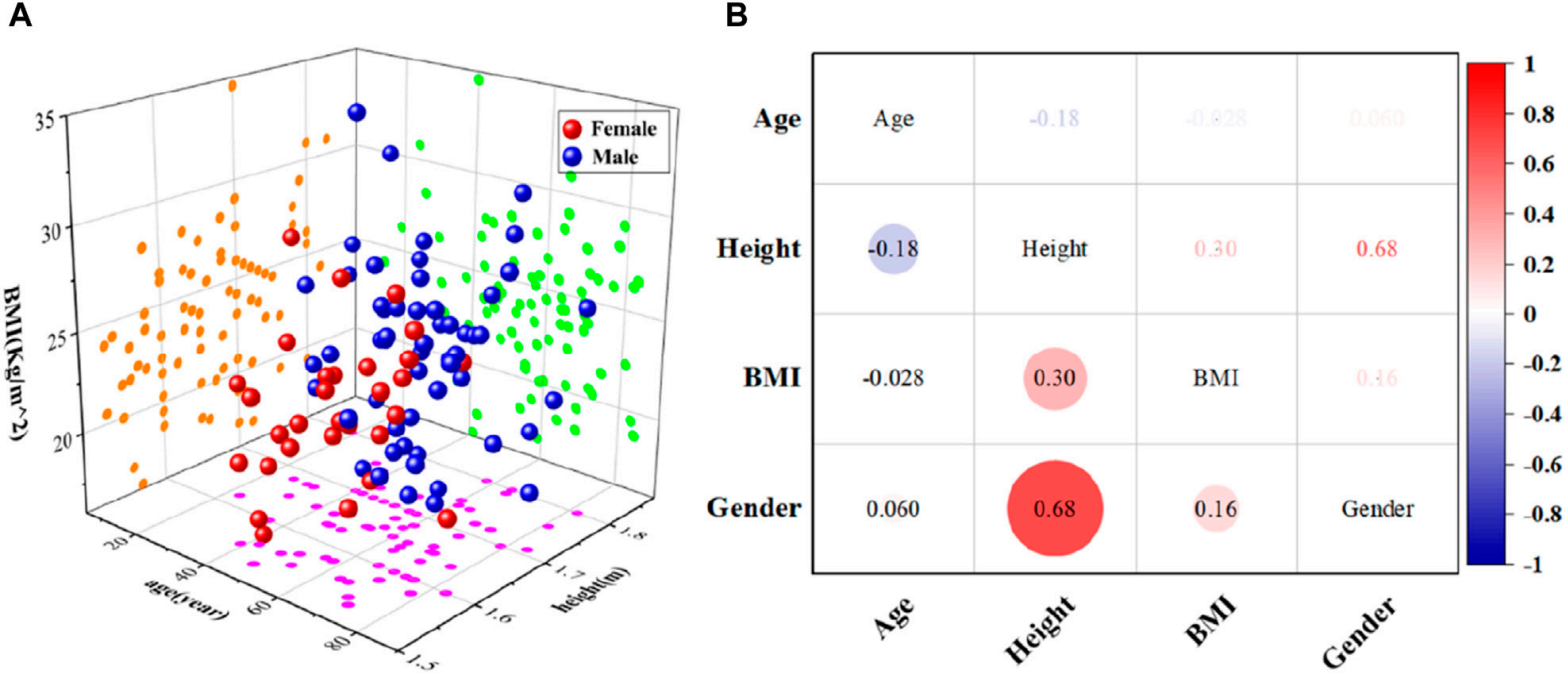
Statistical schematic of head CT scan data samples from different human bodies. **(A)** Distribution of characteristic information corresponding to head CT data samples; **(B)** Heat maps of correlations between age, gender, height and BMI.

Personalized head FE models are necessary in large quantities for studying the response mechanisms of various head anatomical characteristics to head injury. To that purpose, we acquired from Shanghai Ninth People’s Hospital 124 head CT scan data samples from various human bodies. The Shanghai Ninth People’s Hospital authorized this retrospective investigation, and all samples were kept anonymous. There were 61 female and 63 male samples. The samples’ characteristics include age, height, gender, and body mass index (BMI), as shown in Figure 2A. For females, age, height, and BMI ranged from 47.5 ± 35.3 years, 1.61 ± 0.094 m, and 25.4 ± 7.5 kg/m^2^, respectively. For males, age, height, and BMI ranged from 49.1 ± 37.3 years, 1.73 ± 0.174 m, and 24.4 ± 8.5 kg/m^2^, respectively. The parameters of height, age, gender, and BMI are comparatively simple to obtain and can serve as straightforward indicators of the target body’s characteristics. Therefore, these four parameters were universal for personalized human body models. These four parameters had been used in previous research (Hwang et al., 2020; Tang et al., 2020) to create personalized models of various body parts. Figure 2B shows that, other than the significant interaction between height and gender, there is no significant association between the other parameters. This indicates that the four parameters chosen for this study are appropriate for characterizing the head anatomical characteristics. Thus, in this study, the head anatomical characteristics were characterized using age, height, BMI, and gender.

#### 2.1.2 Development and verification of personalized head FE models

**FIGURE 3:**
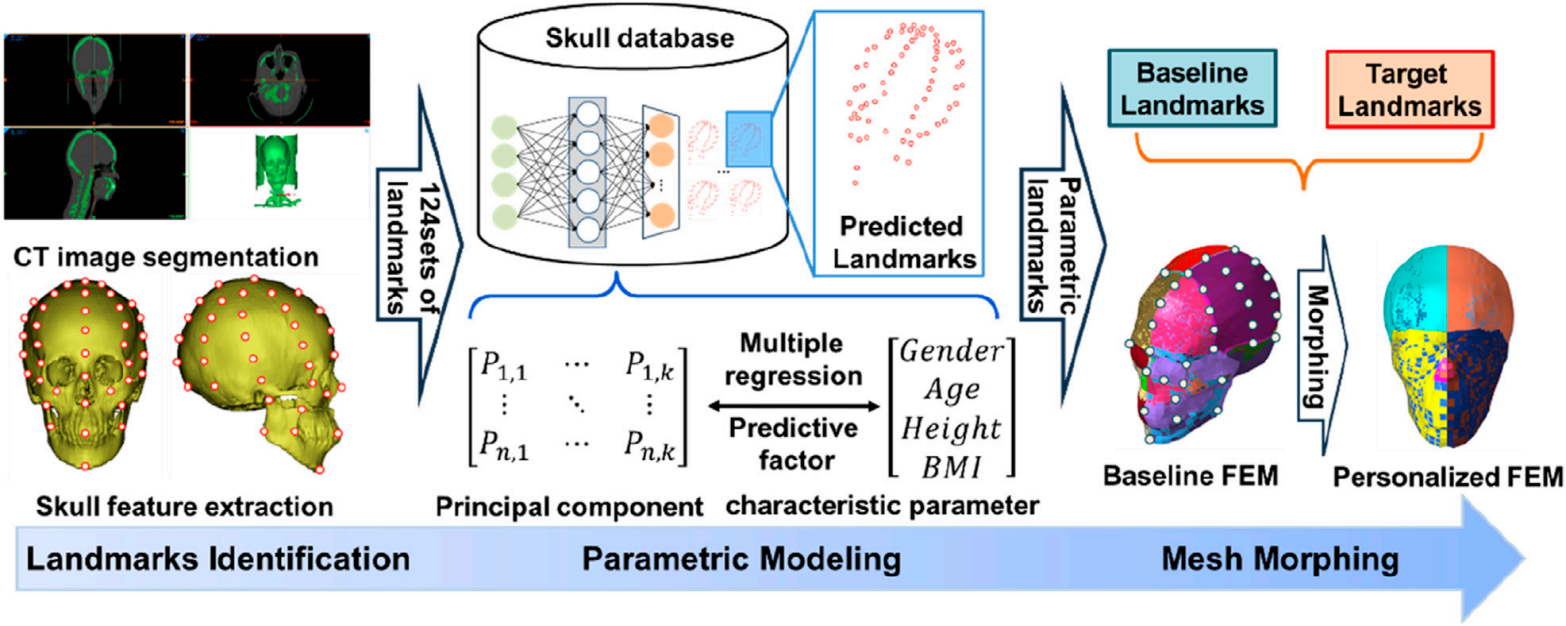
Modeling process of personalized head FE models.

Based on the acquired head CT scan data samples, we developed the personalized head FE models, as shown in Figure 3. First, threshold segmentation and manual area modification were used to get the skull’s geometric surface. 69 landmarks were manually placed on the geometric surface of the skull as target landmarks, while the same number of landmarks were placed at the same locations on the head FE model isolated from the Total Human Model for Safety (THUMS) model as baseline landmarks. Following the dimensional reduction of the three-dimensional coordinate information of the landmarks using Principal Component Analysis (PCA), the regression model of the skull landmarks was created using the characteristic parameters (gender, age, height, and BMI), as indicated by Eq. 1.

$${\left[\begin{array}{ccc} P_{1,1} & \cdots & P_{1,k}\\ \vdots & \ddots & \vdots \\ P_{n,1} & \cdots & P_{n,k} \end{array}\right]}_{n*k}^{T}=A_{k*4}{\left[gender,age,height,BMI\right]}_{4*n}+\varepsilon_{n}$$ (1)

Where, k is the number of principal components after dimensionality reduction of the three-dimensional coordinate information of the landmarks, and k=40 in this study. n is the number of samples, and n=124 in this study. P is the principal component of the three-dimensional coordinate information of the landmarks after dimensionality reduction. A is the regression coefficient matrix. ε is the residual vector.

A total of 200 sets of landmarks were created by entering characteristic parameters using Eq. 1 in order to homogenize the distribution of characteristic parameters in the sample data. Next, using the head FE model that was separated from the THUMS model as the baseline model, we applied the mesh morphing approach based on thin plate spline radial basis function (RBF-TPS) in reference to the research of Hwang et al. (2020) to develop the personalized head FE models. The brain tissue FE model associated with the skull varied with the geometry of the skull based on landmarks on its surface. RBF-TPS details have been thoroughly explained by Tang et al. (2020), and they have been effectively applied to the development of personalized FE models for different human body parts. Finally, distance error statistics between the mesh nodes of the personalized models and the sample geometries were used to verify their geometric accuracy. Statistics on the rate at which the personalized models’ mesh quality changed in comparison to the baseline model were used to verify their mesh quality.

#### 2.1.3 Development and verification of personalized human body MB models

Due to the lack of scanning data from other parts of the body and to improve computing efficiency, we used MB models to develop personalized human body models of different traffic participation states (pedestrian, cyclist). The baseline model, the 50th pedestrian MB model in the MADYMO7.5 (TNO MADYMO BV., Netherlands), has been effectively used in human injury studies across a variety of collision scenarios (Xiao et al., 2020). The model was made up of fifty-two hard parts that depict the human body’s head, neck, chest, abdomen, buttocks, and upper and lower limbs. All the parts were connected together by different types of joints to form a tree-like structure. The personalized human body MB models were obtained by scaling the 50th pedestrian MB model based on the height and BMI characteristics that corresponded to the personalized head FE models. By modifying the arm and leg joint motion, we were able to tie the cyclists MB model to the electric vehicle model.

In this study, the biofidelity of personalized human body MB models was confirmed using data from three cadaver-vehicle crash studies that were taken from published literature (Wu et al., 2017). The scaling approach was used to generate the MB model, which had the same characteristics as the cadaver samples. The vehicle’s design parameters and speed settings match those used in the experiment. In parallel, the correlation between experiment and simulation data was assessed using the correlation and analysis (CORA) approach, which has been applied in related study (Yu et al., 2020), in order to quantitatively assess the differences in the reaction of various head centroid motions in experiment and simulation.

#### 2.1.4 Development of collision scene models

The vehicle MB model, the two-wheeled electric vehicle MB model, and the reduced hood-windshield coupling FE model were all included in the collision scene model used in this study, as shown in Figure 1. The vehicle MB model was Sedan, and the geometric parameters and mass parameters of it come from the National Crash Analysis Center (NCAC). The hood, windscreen, roof, and other parts of the vehicle were modeled using simple ellipsoids. At the same time, the contact characteristics and stiffness between vehicle components were defined based on the published literature (Huang et al., 2019). For the two-wheeled electric vehicle MB model, we referred to the mass and size of two-wheeled electric vehicles established by Gao et al. (2021), which have been verified by real accidents. Handlebars, front fork, front wheel, rear wheel, frame, battery, and seat were the seven rigid bodies that made up the two-wheeled electric vehicle MB model. The contact characteristics and stiffness of the two-wheeled electric vehicle MB model were defined based on the published literature (Huang et al., 2019). The simplified hood-windshield coupled FE model had the same size, mass, and angle parameters as the vehicle MB model. Among these, the hood FE model has an outer layer structure with a thickness of 1.2 mm and an inner layer structure with a thickness of 0.95 mm. The material parameters of the model were derived from the published literature (Shojaeefard et al., 2014; Yu et al., 2017). The windshield FE model was made up of three layers: a 0.76 mm-thick PVB film layer made up the center layer, while the upper and lower glass layers had thicknesses of 2.55 mm and 2.1 mm, respectively.

### 2.2 Development of head injury dataset

According to statistics data from the China In-Depth Accident Study (CIDAS) databases, passenger automobiles most frequently crash with VRU on urban roadways (Chen and Dai, 2018). Among them, vehicles driving at a speed of less than 60 km/h were involved in almost 90% of collisions. Additionally, the VRU injury from the ground was usually greater when the vehicle collides with it at a slow speed (less than 20 km/h). Since the primary focus of this study is the vehicle-VRU collision stage, the vehicle speed during the collision was set between 20 and 60 km/h. Only the impact of speed, the most important influencing factor, on head injury was taken into consideration in this study in order to reduce the overall time cost of the computer CPU. The whole factor experimental design approach for vehicle speed, VRU traffic participation states, and characteristic parameters was used to generate the simulation matrix. Firstly, the location and relative speed of the VRU head in contact with the vehicle were determined in the MADYMO7.5 using the personalized pedestrian MB models, the personalized cyclist MB models, the vehicle MB model, and the two-wheeled electric vehicle MB model. Then, taking the head impact position and relative speed as boundary conditions, we applied the personalized head FE models and simplified hood-windshield coupled FE model to perform simulation calculations in LS-DYNA R8.0 (Livermore Software Technology Corporation, US). Thus, the maximum von Mises value of brain tissue was obtained. Through the above calculation, we obtained the results of 1812 simulation cases. In addition, according to the published literature (Baumgartner et al., 2001; Ho et al., 2006; Yao et al., 2008), the von Mises values obtained were classified into five kinds of head injury degrees: 0–6 kPa was the minor injury; 6–11 kPa was the cerebral contusion; 11–15 kPa was the moderate injury; 15–27 kPa was the cerebral concussion; above 27 kPa was the severe injury.

### 2.3 Development of head injury prediction model

In traffic accidents, the relationship between the VRU injury responses and the feature parameters is extremely nonlinear. Data mining can effectively deal with nonlinear regression and classification problems. Consequently, the prediction of VRU injury responses has made extensive use of ML and DL algorithms. Among these, ML algorithms are capable of predicting results by extracting nonlinear connections from injury datasets. Through the use of a nonlinear multi-layer neural network, the deep learning method is able to predict injury parameters by extracting complex data from the feature parameters. Nonetheless, there are significant differences in the predictive impact of various algorithms across various datasets. We expect to achieve high prediction accuracy by comparing the classification and regression effects of different classical algorithms widely used in injury prediction in the injury dataset established in this study.

Based on the head injury dataset, we applied the ML algorithm, including logistic regression (LR), support vector machines (SVM), decision tree (DT), and random forests (RF), and the DL algorithm, including deep neural network (DNN), convolutional neural network (CNN), long short-term memory (LSTM), and recurrent neural network (RNN), to develop two kinds of injury prediction models: the regression prediction models of head injury response parameter and the classification prediction models of head injury degree. Among these, when creating the regression model, the SVM used for the classification model was converted into the support vector regression (SVR). As for DT, the classification prediction model and regression prediction model were constructed using the classification DT based on the C4.5 algorithm and the regression DT based on the classification and regression trees (CART) algorithm, respectively.

The highest von Mises value of brain tissue was the output of the regression prediction models of head injury response parameter, which also took into account VRU characteristic parameters, traffic participation status, and vehicle speed as inputs. The inputs of the classification prediction models of head injury degree were the same as that of the regression prediction models, and the head injury degree was the output. Simultaneously, the data in the head injury dataset were standardized to increase the models’ accuracy. In this work, 10-fold cross-validation was used to evaluate the prediction model in an effort to increase its accuracy and dependability. The head injury dataset was split up into ten equal-sized sections. One section at a time was randomly chosen as the test set during training, and the remaining nine sections were designated as the training set. The prediction models’ performance index was determined by averaging the value of the loss function after ten iterations. In this work, the regression prediction models’ loss function was the mean square error (MSE) between the predicted von Mises value and the target von Mises value, and the classification prediction models’ loss function was the cross entropy loss between the predicted injury degree and the target injury degree. In addition, we optimized the prediction models’ hyperparameters using a grid search and cross-validation combination. Cross-validation results of prediction model under various hyperparameter combinations were obtained. After that, to construct the final prediction model, the best set of hyperparameters based on the cross-validation results was chosen. The following hyperparameters were chosen for the ML algorithm (regression/classification): 1) LR algorithm’s penalty function was L2, which influences its computational efficiency; 2) SVM algorithm’s kernel function was a Gaussian kernel function with a kernel coefficient of 30/20, which influences its hyperplane definition; 3) DT algorithm’s core algorithm was the C4.5/CART algorithm, which influences its information entropy calculation; and 4) RF algorithm’s number of trees was 10/15, which influences its computational complexity. The DL algorithm in this study were affected by the following hyperparameters: 1) Batch_size, which indicates the quantity of samples used in the training process; 2) Epoch, which indicates the sample’s training times; and 3) Hidden_Size, which indicates the middle layer’s size. Furthermore, the DL-based classification prediction models’ activation function was softmax, whereas the regression prediction models’ activation function was linear. Adaptive moment estimation (Adam) was utilized as the optimizer throughout the training process. The grid search technique results of DL-based prediction models’ hyperparameters were shown in Table 1. Python 3.10 was used to create a model training environment. And the models were trained with an Intel Core i9-10900HQ 2.80 GHz processor.

**TABLE 1:** Parameters of DL algorithm in the regression prediction models of head injury response parameter and the classification prediction models of head injury degree.

Parameters	DNN		CNN		LSTM		RNN	
	Classification	Regression	Classification	Regression	Classification	Regression	Classification	Regression
Epoch	500	300	500	300	400	500	300	400
Batch_size	10	20	10	30	30	10	10	20
Hidden Size	128	128	64	128	64	64	128	64
Optimizer	adam	adam	adam	adam	adam	adam	adam	adam
Loss	categorical_crossentropy	Mse	categorical_crossentropy	Mse	categorical_crossentropy	Mse	categorical_crossentropy	Mse
Activation	softmax	linear	softmax	linear	softmax	linear	softmax	linear

## 3 Results

### 3.1 Performance of personalized models

#### 3.1.1 Performance of personalized head FE models

**FIGURE 4:**
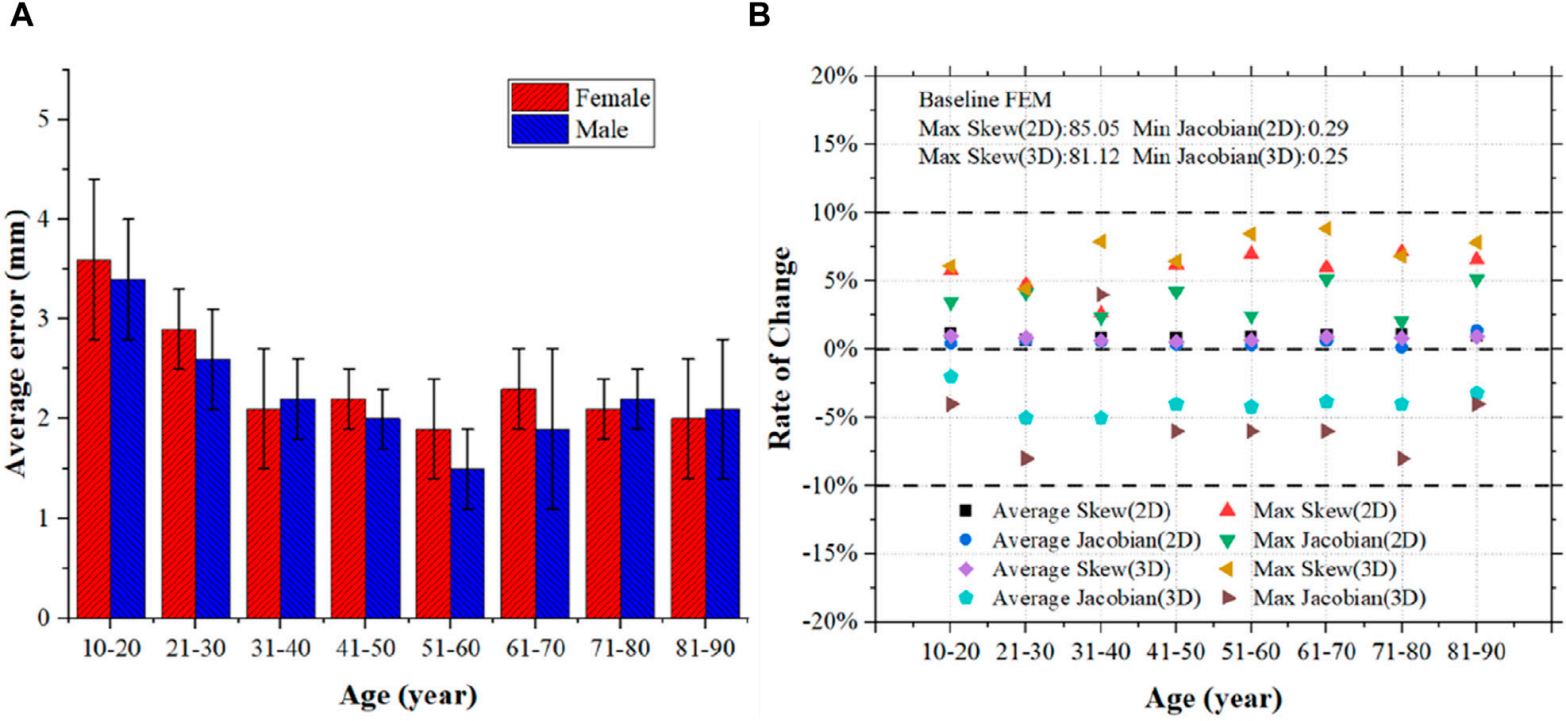
Evaluation of the personalized head FE models. **(A)** Distance error statistics between the mesh nodes of the personalized models and the sample geometries; **(B)** Statistics on the rate at which the personalized models’ mesh quality changed in comparison to the baseline model.

We developed 200 personalized head FE models applying mesh morphing. The geometric error was calculated as the difference between the skull geometric surface retrieved from the matching CT data and the skull model in the established head FE models, as shown in Figure 4A. The personalized head FE models across various age groups have an average geometric error of less than 4 mm and a maximum geometric error of less than 5 mm.

Additionally, the mesh quality of the FE models was assessed using Skew and Jacobian as indicators. The models following mesh morphing modify the mesh quality because of the changes in mesh nodes, as seen in Figure 4B. Among them, the highest change rate is less than 10%, and the average change rate for both 2D and 3D meshes is less than 5%. The generated head FE models have a minimum Jacobian greater than 0.2, while the minimum Jacobian of the 2D and 3D meshes is 0.29 and 0.25, respectively. This demonstrates that the mesh quality of the personalized head FE models may satisfy FE analysis requirements while still maintaining a high degree of consistency with the baseline model.

#### 3.1.2 Performance of personalized human body MB models

**FIGURE 5:**
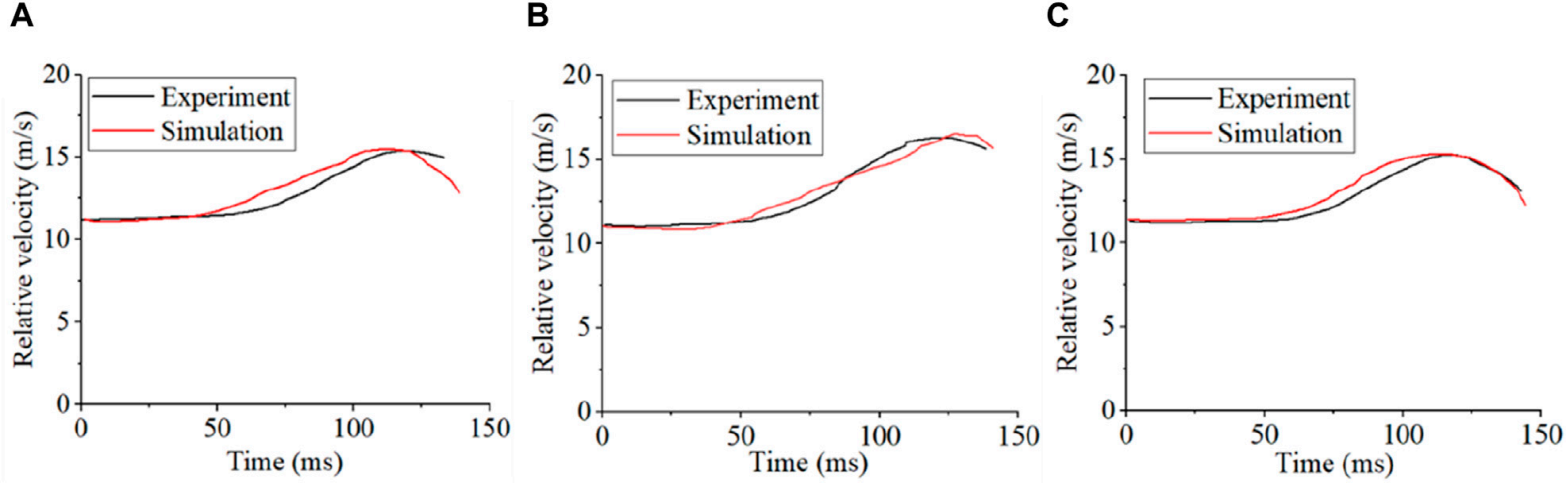
Comparison of head centroid velocity between personalized MB models and cadaver experiment. **(A)** Comparison results between the personalized MB model and V2370; **(B)** Comparison results of personalized MB model and V2371; **(C)** Comparison results of personalized MB model and V2374.

The CORA value pairs of body sample information and head centroid relative velocity with the personalized MB models during a vehicle collision are shown in Table 2. Figure 5 shows a comparison between the head centroid velocity of the personalized MB models and the equivalent cadaveric experiment results. This comparison indicates that the personalized MB models are able to more accurately replicate the cadaveric head motion response during the experiment.

**TABLE 2:** CORA evaluation results of the personalized MB models and the head dynamic response of cadaveric subjects.

Number	Age (year)	Gender	Height(m)	Mass (kg)	Relative velocity CORA (Experiment&Simulation)
V2370	73	Male	1.795	72.6	0.912
V2371	54	Male	1.87	81.6	0.936
V2374	64	Male	1.78	78.0	0.902

### 3.2 Results of head injury dataset

**FIGURE 6:**
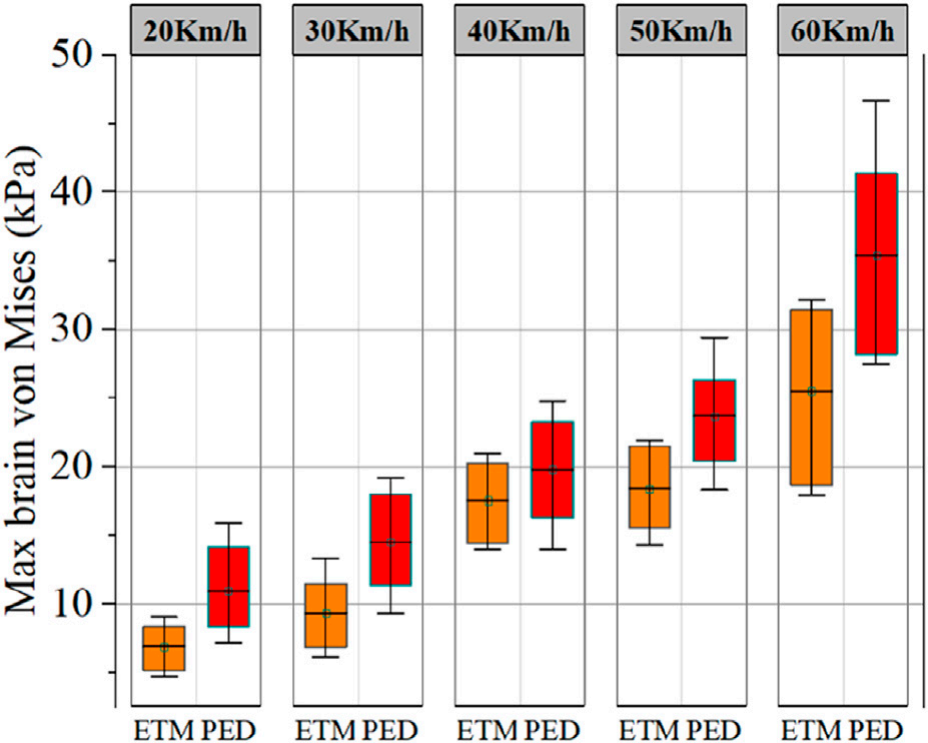
The maximum von Mises values of brain tissue calculated by the personalized head FE models at different collision speeds in the head injury dataset. A 90% confidence interval was shown in the figure as a rectangular region. PED indicates that the VRU state was pedestrian, while ETM indicates that the VRU status was cyclist.

Based on personalized MB and head FE models, 1812 cases were obtained through simulation. Figure 6 displays the highest von Mises value of brain tissue under various working conditions following head-vehicle impacts. As vehicle speed increased, so did the brain tissue’s maximal von Mises value. The von Mises values of VRU brain tissue are between 4.4 and 46.9 kPa at speeds ranging from 20 to 60 km/h. Furthermore, the average maximum von Mises values of brain tissues are higher for pedestrians than for cyclists.

**FIGURE 7:**
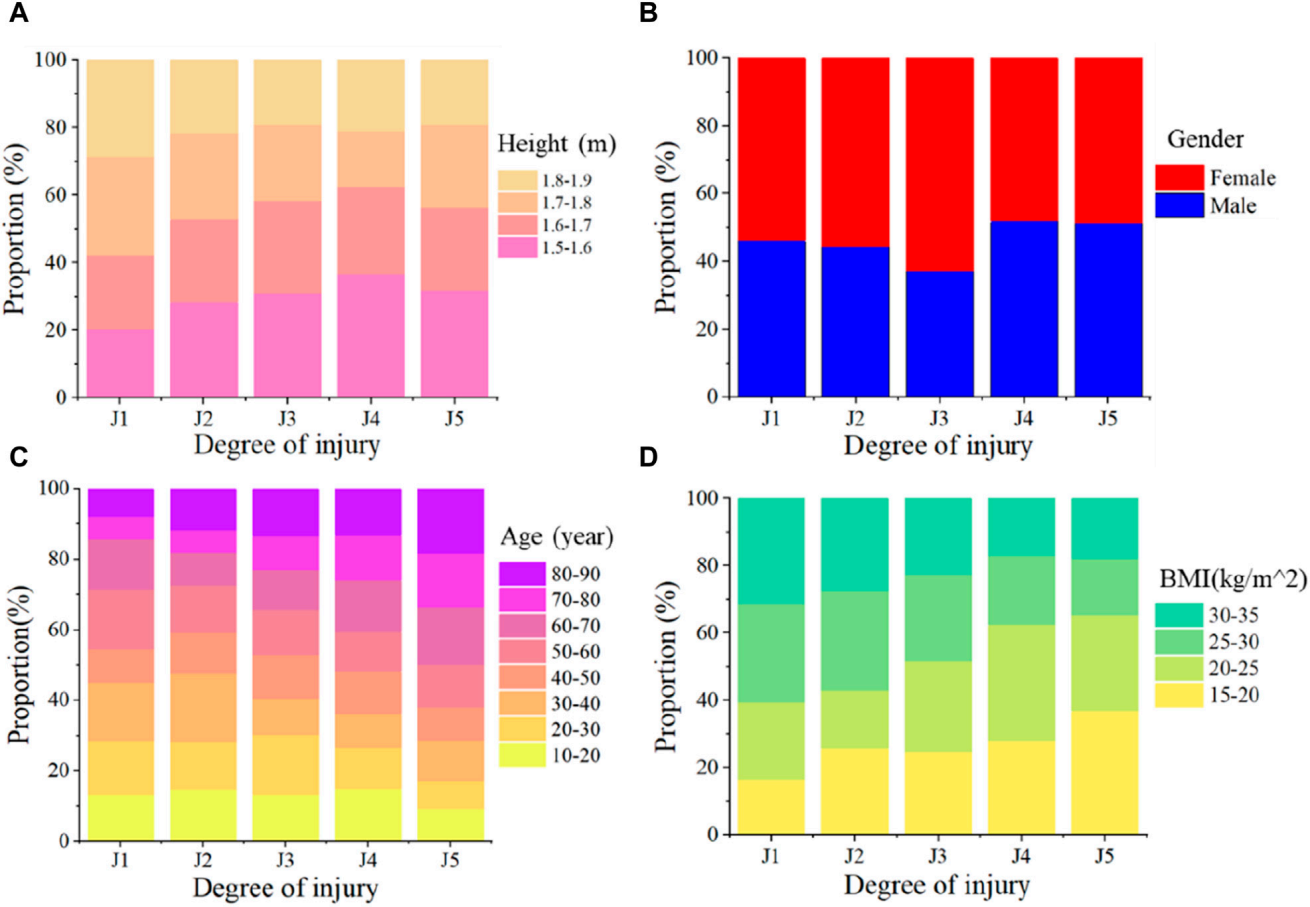
Statistical results of the influence of different human characteristics parameters on the degree of VRU head injury in traffic accidents. The symbols J1, J2, J3, J4, and J5 represent different levels of injury: minor injury, cerebral contusion, moderate injury, cerebral concussion, and severe injury. **(A)** The effect of height on the degree of head injury; **(B)** The effect of gender on the degree of head injury; **(C)** The effect of age on the degree of head injury; **(D)** The effect of BMI on the degree of head injury.

The impact of various human characteristics parameters on the head injury degree of VRU in traffic accidents was statistically represented in Figure 7, where J1, J2, J3, J4, and J5 represent, respectively, minor injury, cerebral contusion, moderate injury, cerebral concussion, and severe injury. The impact location causes a high degree of nonlinearity and uncertainty in the relationship between height and head injury. For VRU with a height of under 1.7 m, the probability of a higher level of head injury increases by an average of 4% at every level for J1–J4 levels, whereas the probability of suffering a serious injury is the same for VRU over 1.7 m and those below 1.7 m, as seen in Figure 7A. Head injury is not significantly impacted by the VRU’s gender when only the geometric anatomical structure of the head is taken into account, as Figure 7B illustrates. As shown in Figure 7C, the risk of higher levels of head injury gradually increased with age, and the risk of severe injury in a traffic accident is 10% higher in VRU over 80 than in VRU between the ages of 10 and 20. VRU with a higher BMI have a decreased risk of higher head injury, as Figure 7D illustrates, and an 8% increased risk of severe injury in a traffic accident is associated with VRU with a BMI of 15–20 kg/m^2^ compared to 30–35 kg/m^2^.

### 3.3 Results of head injury prediction model

**FIGURE 8:**
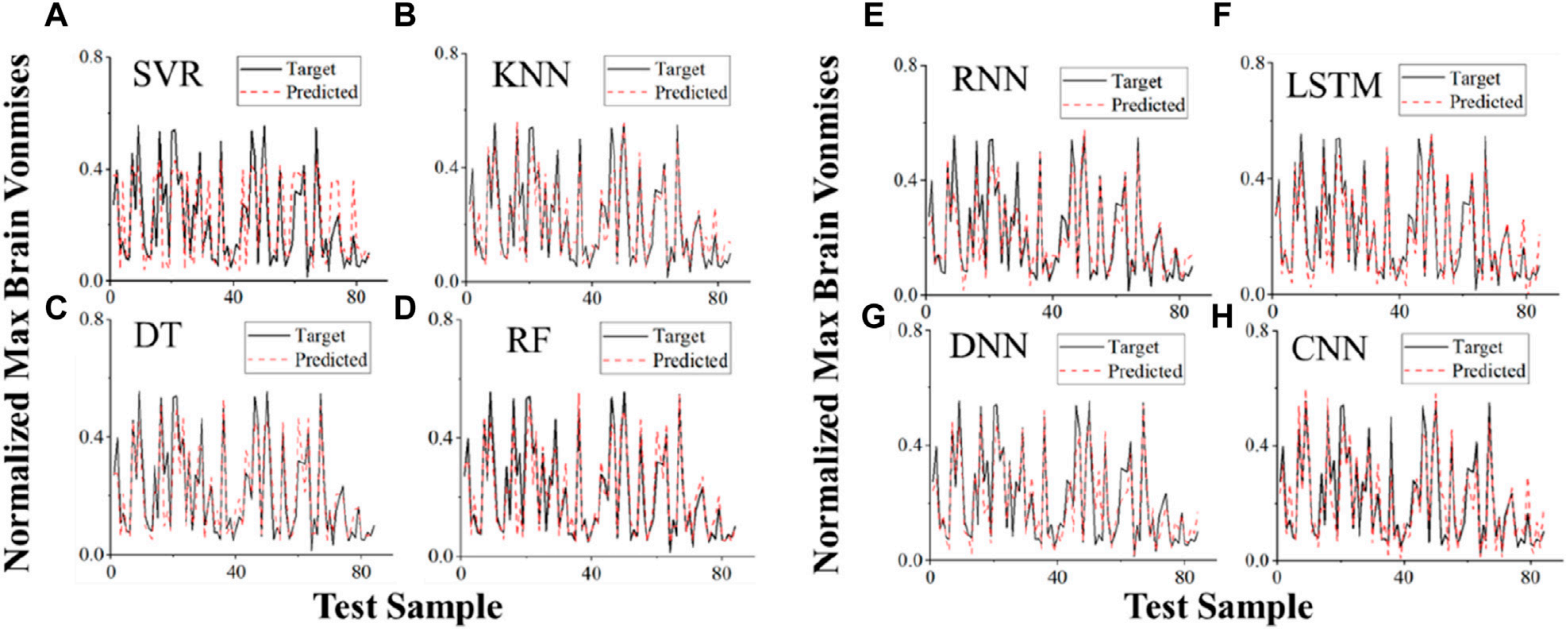
Examples of using different regression prediction models of the head injury response parameter to predict normalized von Mises values in the test dataset. **(A)** SVR-based model; **(B)** KNN-based model; **(C)** DT-based model; **(D)** RF-based model; **(E)** RNN-based model; **(F)** LSTM-based model; **(G)** DNN-based model; **(H)** CNN-based model. In head injury regression prediction, SVR, DT, and RF all show similar results, with R2 values of 0.74, 0.75, and 077, respectively. RNN and LSTM frequently produce outstanding outcomes when a sequence of input feature parameters exists. Nevertheless, since the characteristic parameters were discretized in this study, RNN and LSTM are ineffective for predicting head injury regression.

Applying four ML algorithms (SVM, LR, DT, RF) and four DL algorithms (RNN, LSTM, DNN, CNN), eight different types of regression prediction models of the head injury response parameter and the classification prediction models of the head injury degree were developed based on the head injury dataset. As shown in Table 3, the R2 value, mean square error (MSE), and mean absolute error (MAE) of the DL-based models are higher than those of the ML-based models. Among them, the DNN algorithm has the best performance, compared with the RF algorithm, which has the best performance in ML. In the R2 value, MSE and MAE were 0.85 vs. 0.77, 0.006 vs. 0.014, and 0.055 vs. 0.073, respectively. In the ML-based models, LR is difficult to deal with due to the high degree of uncertainty and nonlinearity in traffic accidents, so the regression prediction ability is poor, and the R2 value is only 0.54. Additionally, as the normalized von Mises value prediction example in Figure 8 illustrates, there is a significant deviation between the predicted and actual values of the prediction model using the LR algorithm.

**TABLE 3:** Performance comparison of four ML-based models and four DL-based models in the regression prediction of von Mises value.

Indicator	ML				DL			
	SVR	KNN	DT	RF	DNN	CNN	LSTM	RNN
R2	0.54	0.69	0.65	0.71	0.85	0.81	0.69	0.67
MSE	0.026	0.018	0.021	0.014	0.006	0.011	0.018	0.021
MAE	0.092	0.062	0.078	0.073	0.055	0.061	0.058	0.061

In terms of head injury degree prediction, as shown in Table 4, the DL-based models and the ML-based models perform comparably. All the DL-based models and ML-based models anticipate AUC values above 0.8, with the exception of the LR algorithm, which is not appropriate for handling nonlinearized data. The SVM algorithm outperforms all other algorithms in terms of accuracy and AUC, with values of 0.902 and 0.87, respectively.

**TABLE 4:** Performance comparison of four ML-based models and four DL-based models in the classification prediction of head injury degree.

Indicator	ML				DL			
	SVM	LR	DT	RF	CNN	CNN	LSTM	RNN
Accuracy	0.89	0.59	0.71	0.72	0.86	0.854	0.815	0.792
Recall	0.76	0.59	0.75	0.76	0.75	0.718	0.695	0.688
F-score	0.76	0.58	0.74	0.76	0.75	0.71	0.695	0.612
AUC	0.902	0.645	0.744	0.753	0.872	0.856	0.843	0.829

## 4 Discussion

### 4.1 The influence of anatomical characteristics on VRU head injury

The von Mises value of brain tissue covers a wide range at various speeds, as seen in Figure 6. This is due to the fact that head injury is likewise significantly influenced by body sizes and head anatomical characteristics. In terms of the height factor, head acceleration during collisions was found to gradually decrease with increasing height, as reported by Sankarasubramanian et al. (2016) and Huang et al. (2019). However, only the head’s dynamic response during the accident was taken into account in the above-mentioned study. Compared to the windshield, a high von Mises value was frequently produced when the head FE model collided with the hood, according to the study of Huang et al. (2018). Therefore, as shown in Figure 7A, the height factor exhibits a high degree of nonlinearity and uncertainty in head injuries. Regarding the gender factor, as shown in Figure 7B, this study finds that it has no discernible effect on head injuries. This finding is in line with Huang et al. (2018)’s investigation results of actual accidents. Regarding the age factor, as shown in Figure 7C, the head injury of VRU in traffic accidents progressively worsens with age, which is in line with the findings of the research on actual accidents carried out by Feng et al. (2020). But in this study, the degree of injury rises a little with age. This is because, while developing the personalized head FE models, the study only took geometric anatomy into account, neglecting material concerns. Regarding the BMI factor, as seen in Figure 7D, this study finds that those with higher BMIs experience fewer head injuries in accidents. This is because the body with a higher BMI has a bigger moment of inertia under the same collision torque, which causes the head to accelerate comparatively little.

### 4.2 The performance of the head injury prediction model

Due to the linear structure of the algorithm’s inability to handle the uncertainty and nonlinear relationship between the factors, as can be shown in Tables 3, 4, the LR algorithm performs the worst in both regression and classification prediction. The DL algorithm performs the best regression prediction ability (R2: 0.85, MSE: 0.006, MAE: 0.055). This is due to the fact that the DL algorithm has the ability to build intricate hidden layer networks that handle nonlinear relationships between factors. Yet due to the application of time-step based series input for feature input, LSTM and RNN algorithms are better suited for processing continuous data, albeit they have a little inferior capacity to predict regression. For instance, Wang Q. F. et al. (2021) observed that the algorithm used in conjunction with RNN-CNN allowed for an accurate prediction of the head acceleration curve, with a R2 value of 0.76. Nonetheless, there is no clear continuity association between the von Mises values and the factors in this study, which includes human characteristics parameters, vehicle speed, and traffic participation status. Considering the classification prediction models of head injury degree, Table 4 shows that the majority of the DL-based models outperform the ML-based models in prediction. The highest result is, however, obtained by the SVM algorithm prediction ability (Accuracy: 0.89, Recall: 0.76, F-score: 0.76, AUC: 0.902). This is so that discrete factors in small sample dataset may be processed effectively using the hyperplane technique, which is the foundation of the SVM algorithm. Given that the study’s discrete data include both human gender and traffic participation states, which were represented by the algorithm construction as 0/1, the SVM hyperplane can produce a better classification result. Wang Q. F. et al. (2021) constructed the dataset using a 0/1 representation for both human gender and seat belt use, and they also achieved the best classification prediction effect using the SVM algorithm. In addition, the binary classification problem has a comparatively low prediction complexity in terms of classification prediction. For example, Delen et al. (2017) and Lamba et al. (2019) built the human injury prediction models with high/low injury degree classification based on the SVM algorithm, and the accuracies were 0.904 and 0.966, respectively. The prediction category in this study is five, which makes it more difficult to predict than a binary classification problem. Despite this, the SVM algorithm’s prediction accuracy can still reach 0.89, demonstrating its superiority in handling head injury classification prediction.

### 4.3 Real-world relevance of the study

Future vehicle safety technology development will mostly follow the path of active and passive safety integration. In this study, the head injury state and von Mises value of VRU with different anatomical characteristics were obtained, and the corresponding prediction model was developed. In terms of passive safety, this research can serve as a guide for optimizing the design of vehicles’ structures, strategically placing bumpers, and utilizing other safety measures to effectively protect VRU with particular anatomical characteristics in potential collisions. For active safety, the prediction model developed in this study can be used to predict the VRU injury caused by an impending collision in advance and provide a basis for specific vehicle active decision control for VRU with different anatomical characteristics. Furthermore, the study’s findings can serve as a guide for pertinent regulatory organizations and institutions as they create guidelines and standards for VRU collision protection with various anatomy characteristics.

### 4.4 Limitations and future work

There are several limitations to the study. First, only the geometric anatomical characteristics were taken into account when the personalized head FE models were developed, and the changes in the head material parameters with respect to characteristic parameters were not taken into account. The impact of material factors on injury results in personalized head FE models will be examined in further study. Second, due to time constraints, when developing the VRU head injury dataset, in addition to considering the geometric and anatomical characteristics of VRU itself, we only considered the vehicle speed and the traffic participation status of VRU. It is demonstrated that the dynamic reaction characteristics of the head during a collision are directly influenced by the type of vehicle and collision angle. Thus, it will be appropriate to develop a VRU head injury dataset in the future that takes into account more factors. In addition, the processing capacity of various algorithms for head injury data varies, thus future study will concentrate on how to use algorithm coupling techniques (e.g., RNN-CNN, DNN-LSTM, etc.) to increase the precision and effectiveness of injury prediction.

## 5 Conclusion

This study proposed a data-mining-based methodology for predicting VRU head injuries, taking into account body sizes and head anatomical characteristics. The objective of the present study is to investigate the influences of anatomical characteristics on the VRU head injury in collision incidents and develop a prediction model that can accurately predict it. The framework consists of three parts: 1) Based on mesh morphing and scaling, respectively, personalized head FE models and human body MB models were developed; 2) A head injury dataset was created, where the dependent variables were the degree of head injury and the von Mises value of brain tissue, and the independent variables were VRU’s height, BMI, age, gender, traffic participation states, and vehicle speed. 3) Eight kinds of data mining algorithms (SVM, LR, DT, RS, RNN, LSTM, DNN, and CNN) were applied to develop the regression prediction models of the head injury response parameter and the classification prediction models of the head injury degree. After testing, the best R2 value of the regression prediction model is 0.85, and the best accuracy rate of the classification prediction model is 0.89. Through the head injury prediction framework, the following conclusions can be drawn.

(1) The von Mises value of VRU brain tissue during collision ranges from 4.4 kPa to 46.9 kPa at speeds between 20 and 60 km/h, with cyclists having an average von Mises value that is marginally lower than pedestrians.
(2) The risk of head injury increases with the VRU’s age, and the risk of severe injury in a traffic accident is 10% higher in VRU over 80 than in VRU between the ages of 10 and 20; VRU with a higher BMI have fewer head injuries in collision accidents, and an 8% increased risk of severe injury in a traffic accident is associated with VRU with a BMI of 15–20 kg/m^2^ compared to 30–35 kg/m^2^; VRU’s height had a highly erratic and nonlinear impact on head injury; and head injury is not significantly impacted by the VRU’s gender.
(3) The DL algorithm performs better in the regression prediction models of the head injury response parameter because it can handle the nonlinear and ambiguous relationship between factors. In addition, the SVM algorithm performs better in classification and prediction when the head injury dataset contains discrete data (e.g., gender, traffic participation status, etc.).

## Data Availability

The original contributions presented in the study are included in the article/supplementary material, further inquiries can be directed to the corresponding author.
